# Characterization of Adipose-Derived Stem Cells Following Burn Injury

**DOI:** 10.1007/s12015-017-9721-9

**Published:** 2017-06-23

**Authors:** Anesh Prasai, Amina El Ayadi, Randy C. Mifflin, Michael D. Wetzel, Clark R. Andersen, Heinz Redl, David N. Herndon, Celeste C. Finnerty

**Affiliations:** 1 Cell Biology Graduate Program, University of Texas Medical Branch, Galveston, TX USA; 2 Department of Surgery, University of Texas Medical Branch, Galveston, TX USA; 3 Shriners Hospitals for Children®—Galveston, Galveston, TX USA; 4 Ludwig Boltzmann Institute for Experimental and Clinical Traumatology, Vienna, Austria; 5 Institute for Translational Sciences and Sealy Center for Molecular Medicine, University of Texas Medical Branch, 301 University Blvd., Galveston, TX 77555-1220 USA

**Keywords:** Adipocytes, Adipose-derived stem cell, Adipose tissue, Burns, Inflammation, Stromal vascular fraction

## Abstract

Severe burns induce a prolonged inflammatory response in subcutaneous adipose tissue that modulates signaling in adipose-derived stem cells (ASCs), which hold potential for healing burn wounds or generating skin substitutes. Using a 60% rat scald burn model, we conducted a series of experiments to determine which cells isolated from the adipose tissue produced inflammatory mediators and how these changes affect ASC fate and function. The stromal vascular fraction (SVF), adipocytes, and ASCs were isolated from adipose tissue at varying times up to 4 weeks postburn and from non-injured controls. Endpoints included inflammatory marker expression, expression of ASC-specific cell-surface markers, DNA damage, differentiation potential, and proliferation. Inflammatory marker expression was induced in adipocytes and the SVF at 24 and 48 h postburn; expression of inflammatory marker mRNA transcripts and protein returned to normal in the SVF isolated 1 week postburn. In enriched ASCs, burns did not alter cell-surface expression of stem cell markers, markers of inflammation, differentiation potential, or proliferative ability. These results suggest that adipocytes and the SVF produce large quantities of inflammatory mediators, but that ASCs do not, after burns and that ASCs are unaffected by burn injury or culturing procedures.. They also suggest that cells isolated over 48 h after injury are best for cell culture or tissue engineering purposes.

## Introduction

In 2013 alone, 35 million burn injuries were reported worldwide and resulted in approximately 2.9 million hospitalizations and 238,000 deaths [1, 2]. In patients with large burns, wound coverage options are limited, mainly due to the lack of donor sites. New approaches are needed to cover massive burn wounds. Over the past decade, the discovery of adult mesenchymal stem cells in a plethora of tissues has enabled development of stem cell-based therapies for clinical use [2]. Stem cells isolated from adipose tissue (adipose-derived stem cells or ASCs) are similar to bone marrow-derived mesenchymal stem cells (MSCs) in that ASCs can differentiate into many cell types including chondrocytes, osteoblasts, adipocytes, cardiomyocytes, endothelial cells, epithelium, and neuronal cells. Furthermore, it is relatively easy to isolate up to 5000 ASCs from one gram of adipose tissue [3–5]. Aside from their ability to differentiate into a variety of cell types, ASCs also secrete an abundance of cytokines and growth factors, including interleukin (IL)-6, IL-7, IL-8, IL-11, IL-12, fibroblast growth factor, epidermal growth factor, keratinocyte growth factor, and macrophage colony-stimulating factor [6, 7]. The production of these factors may account for the therapeutic effect of ASCs in wound healing [8, 9], tissue regeneration [10], angiogenesis [11], and immunomodulation [9].

We and others have proposed that ASCs can be easily obtained from the subcutaneous adipose tissue that is discarded during debridement surgeries following a severe burn injury and that these ASCs can be used for wound healing or tissue regeneration [12–14]. Additional opportunities for ASC isolation are present during subsequent operations that massively burned patients must undergo and uncover other subcutaneous fat depots. Because coverage options for large burn wounds are limited, autologous ASCs could be applied to severely burned patients to facilitate wound closure; this could be accomplished either directly after harvest in the form of the stromal vascular fraction (SVF) or following manipulation of the enriched ASCs in culture to generate replacement grafts. We have recently shown that a severe burn injury induces a prolonged inflammatory response in subcutaneous adipose tissue isolated from unburned regions [15, 16]. The effects of this inflammatory environment on ASCs are of concern, as inflammatory conditions such as diabetes have been shown to affect the basic properties of MSCs; when transplanted after myocardium infarction, MSCs from diabetic patients have a lower proliferative capacity and a weaker myocardial protective effect than MSCs from non-diabetic patients [17]. Studies on many different types of stem cells have revealed that the fate and function of a stem cell are controlled by factors in the microenvironment of the surrounding niche [18, 19].

Severe burn injury induces a systemic hypermetabolic and inflammatory response that results in elevation of stress hormones and inflammatory cytokines, which can last for several years. Long-term perturbations also occur in non-burned muscle, fat, and skin [20]. In adipose tissue, these responses can last for at least a year [20]. As a result, ASCs that reside at the burn site, those that migrate to the burn site through the circulatory system, and those residing in adipose depots far away from the burn injury are all exposed to altered levels of cytokines and hormones, as well as growth factors secreted in response to the burn injury. As the niche surrounding the stem cells defines the characteristics of these stem cells and can direct cell function and fate [19], we designed this study to determine which cells within the adipose tissue contributed toward the production of inflammatory mediators and whether ASCs are affected by the post-burn inflammatory environment. Using a 60% rat scald burn model, we analyzed inflammatory responses of adipocytes, the SVF (comprised of multiple cell types including ASCs, inflammatory cells, and pre-adipocytes), and enriched ASCs. Endpoints studied in the ASCs included differentiation potential, proliferation, cell surface cluster of differentiation and inflammatory marker expression, and DNA damage. We also determined whether the time of adipose collection postburn yields ASCs with differing properties, as much of our previous work shows a temporal pattern for burn-induced elevation of inflammatory mediators both systemically and within adipose tissue.

## Materials and Methods

### Rat Model of Burn Injury

All animal experiments adhered to the guidelines detailed in the NIH Guide for the Care and Use of Laboratory Animals. The study was reviewed and approved by the Institutional Animal Care and Use Committee of the University of Texas Medical Branch (Galveston, TX). Male Sprague–Dawley rats weighing 250 g were housed in an animal facility with a 12-h light and dark cycle for 1 week to acclimate prior to the initiation of the experiment. All animals received water and food ad libitum and were monitored for the entire study period. Eight animals were included in the control group, while 6 animals were included in each burn group. Prior to the burn injury, 0.05 mg/kg buprenorphine hydrochloride was administered as analgesia, followed by 40 mg/kg ketamine with 5 mg/kg xylazine (IP) as anesthesia. A 60% total body surface area scald burn was introduced in the manner previously described [21, 22]. Resuscitation was accomplished by intraperitoneal injection of 60 mL/kg Ringer’s lactate solution. Buprenorphine hydrochloride was administered every 12 h to relieve discomfort. Rats were euthanized at 24 h or 48 h or at 1, 2, or 4 weeks following burn injury. Subcutaneous adipose tissue (0.9–3.0 g) was obtained from burned rats and sham-treated control rats for isolation of adipocytes, SVF, and/or ASCs at these time points.

### ASC Isolation

Following its removal, adipose tissue was washed extensively with phosphate buffered saline (PBS) containing 5% penicillin/streptomycin. The tissue was then minced and incubated with 0.075% collagenase Type IA at 37 °C for 60 to 80 min with constant shaking. An equal volume of complete media (Dulbecco’s Modified Eagle Medium, 10% fetal bovine serum, and 2% antibacterial/antimycotic solution [10,000 IU/mL penicillin, 10,000 μg/mL streptomycin, 25 μg/mL amphotericin, 8.5 g/L sodium chloride]) was used to inactivate the collagenase. The solution was aspirated and centrifuged at 350 g for 5 min to separate the cells from the adipose tissue. The layer floating on the top was composed of mature adipocytes; these cells were removed and stored in RNA lysis buffer until analysis by real time polymerase chain reaction (PCR). The pellet at the bottom of the tube was the SVF, comprising endothelial cells, fibroblasts, ASCs, immune cells, and other cells. This SVF pellet was reconstituted with PBS and centrifuged at 350 g for 5 min. This step was repeated 3 to 4 times until the supernatant became clear. The pellet was washed with water, and 10X PBS was added to lyse the red blood cells. After these steps, the pellet was resuspended in complete media and filtered through a 70-μm cell strainer. The resulting mixture was divided into two aliquots. The first half was labeled SVF; these cells were preserved in RNA lysis buffer and stored for later analysis. The remaining aliquot was plated on two 100-cm dishes to isolate enriched ASCs. After an 18 h incubation, the media was aspirated to remove any unattached cells and debris, and complete media was added to the culture dishes. Following 30 additional hours of incubation, one plate of enriched ASCs was treated with RNA lysis buffer, and the lysate was stored for further analysis. The ASCs in the remaining plate were expanded in culture until the fourth passage was reached, and the cells were then used for further experimentation.

### Differentiation

ASCs were grown for 4 weeks in complete media with the following components added to induce differentiation into each indicated cell type.

- Osteogenic cells: 0.1 μM dexamethasone, 50 μM ascorbate-2-phosphate, 10 mM β-glycerolphosphate, 0.1 μM retinoic acid
- Adipogenic cells: 1 μM dexamethasone, 10 μM insulin, 0.5 mM isobutyl-methylxanthine, 200 μM indomethacin
- Chondrogenic cells: 6.25 μl/mL insulin, 10 ng/mL TGF-β, 50 nM ascorbate-2-phosphate, 2% fetal bovine serum
- Epithelial cells: 10 μM all-trans retinoic acid

Cells were then harvested and fixed for histology or immunocytochemistry. Alternatively, RNA was isolated for real time PCR. Osteogenic cells were stained using alizarin red, adipogenic cells with oil-O-Red, and chondrogenic cells with alcian Blue. Epithelial cells were subjected to immunohistochemistry for CK-14. Nuclei were visualized with 4′,6-diamidino-2-phenylindole.

### Real-Time PCR

Total RNA was isolated using the RNeasy mini kit (Qiagen, Chatsworth, CA) according to the manufacturer’s instructions. RNA was quantified using the NanoDrop method (NanoDrop Technologies, Wilmington, DE); reverse transcription reactions were performed with 500 ng of total RNA using the iScript cDNA synthesis kit (Bio-Rad, Laboratories, Hercules, CA). Real-time PCR was performed using SYBR green, while the Step One plus real time PCR system (ThermoFisher Scientific, Waltham, MA) was used for amplification and data collection. PCR conditions were as follows: 95 °C for 10 min, 40 cycles at 95 °C for 15 s, and 55 °C for 30 s. The primer sequences are listed in Table 1. The delta delta CT method was used to quantify gene expression, which was then normalized to expression of the internal housekeeping genes, glyceraldehyde 3-phosphate dehydrogenase (GAPDH) and cyclophilin A.

**Table 1:** Primers for CD marker characterization, differentiation, and inflammation

Gene	Forward Primer	Reverse Primer
Adiponectin	5′-AATCCTGCCCAGTCATGAAG-3′	5′-GTCCCCTTCCCCATACACTT-3′
ALPB-1	5′-TAAGGGTGACCCAGGAGATG-3′	5′-GGAACATTGGGGACAGTGAC-3′
Osteonectin	5′-CTGCCACTTCTTTGCGACCA-3′	5′-CTCCAGGCGCTTCTCGTTCTC-3′
Osteopontin	5′-CTGGCAGTGGTTTGCCTTTGC C-3′	5′-CGTCAGATTCATCCGAGTTCAC-3′
ChM1	5′-GTGGTCCCACAAGTGAAGGT-3′	5′-TCGACCTCCTTGGTAGCAGT-3′
Collagen II	5′-GAACAACCAGATCGAGAGCA-3′	5′-CTCTCCAAACCAGATGTGCT-3′
CD11b/c	5′-CTGGGAGATGTGAATGGAG-3′	5′-ACTGATGCTGGCTACTGATG-3′
CD73	5′-TCAAATCTGCCTCTGGAAAG-3′	5′-TTCCCCTACCCACTACCTTC-3′
CD90	5′-AGCCAGATGCCTGAAAGAGA-3′	5′- TGATAGAAGGGGGCTGAGAA-3′
CD34	5′-TCTTGGCCAATAGCACAGAACT-3′	5′-TGCAATCAGAGTCTTTCGGGAA-3′
CD105	5′-CTGGAGCAGGGACGTTGT-3′	5′-GCTCCACGCCTTTGACC-3′
Cycophilin A	5′-TATCTGCACTGCCAAGACTGAGTG-3′	5′-CTTCTTGCTGGTCTTGCCATTCC-3′
CK-10	5′-TGGTTCAATGAAAAGAGCAAGGA-3′	5′-GGGATTGTTTCAAGGCCAGTT-3′
CK-14	5′-GGCCTGCTGAGATCAAAGACTAC-3′	5′-CACTGTGGCTGTGAGAATCTTGTT-3′
IL-1ß	5′-CACCTTCTTTTCCTTCATCTTTG-3′	5′-GTCGTTGCTTGTCTCCTTGTA-3′
IL-6	5′-CGAGCCCACCAGGAACGAAAGTC-3′	5′-CTGGCTGGAAGTCTCTTGCGGAG-3′
Caspase-1	5′-CACATTGAAGTGCCCAAGCT-3′	5′-TCCAAGTCACAAGACCAGGC-3′
MCP-1	5′-GTTGTTCACACTTGCTGCCT-3′	5′-CTCTGTCATACTGGTCACTTCTAC-3′
NF-κB	5′-GTGCAGAAAGAAGACATTGAGGTG-3′	5′-AGGCTAGGGTCAGCGTATGG-3′
TNF-α	5′-TCAGCCTCTTCTCATTCCTGC-3′	5′-TTGGTGGTTTGCTACGACGTG-3′

### Flow Cytometry

Fourth passage ASCs were cultured in complete media until sub-confluent prior to flow cytometric analysis (Becton Dickinson FACSCanto cytometer, Franklin Lakes, NJ). Media was removed by washing the ASCs with PBS. The ASCs were then harvested with 0.25% trypsin/EDTA. Cell viability was assessed via propidium iodide staining. Determination of fluorescent cells, dead cells, debris, and background noise was made with FACS DIVA software (Becton Dickinson, Franklin Lakes, NJ). For extracellular staining, the cells were treated with a mixture of ice-cold 1% sodium azide for 30 min, washed with PBS, and incubated with 3% bovine serum albumin (BSA) for 30 min on ice. Following a PBS wash, aliquots of ASCs were incubated in BSA with the following monoclonal antibodies for flow cytometry: CD73-Alexa 488 (BD Biosciences, San Jose, CA), CD90-Alexa 488 (BioLegend, San Diego, CA), CD11b-Alexa 488 (BioLegend, San Diego, CA), CD34-PE (Santa Cruz Biotechnology, Dallas, TX), CD36-Alexa 488 (BioLegend, San Diego, CA), and CD29-PECy7 (BioLegend, San Diego, CA). For intracellular staining, cells were counted and fixed with 4% paraformaldehyde for 20 min at room temperature, washed three times with PBS, and lysed with 90% ice-cold methanol for 30 min. The lysed cells were incubated in 3% BSA for 30 min and treated with a monoclonal primary antibody to CD105-PE (Bioss Inc., Woburn, MA).

### Proliferation

Cells were plated at a density of 5000 cells per well in xCELLigence® plates (ACEA Biosciences, San Diego, CA). Cellular attachment, spreading, and proliferation were monitored every 15 min using the real-time cell electronic sensing (RT-CES)® system (ACEA Biosciences, San Diego, CA), which measures cellular proliferation based on impedance. Cell-sensor impedance was expressed as an arbitrary unit called the Cell Index. Data were recorded for 55 h and then analyzed.

### Alkaline Single Cell Micro Gel Electrophoresis (Comet) Assay

Comet assays were performed using the OxiSelect Comet Assay Slides according to the manufacturer’s instructions (Cell Biolabs Inc., San Diego, CA). A cellular suspension in PBS (1 × 10^5^ cells per mL) was mixed 1:10 with molten low-melting-point agarose at 37 °C. Seventy-five microliters of this suspension were then placed within each well on the specially prepared microscope slide provided with the kit. Slides were incubated at 4 °C for 15 min to allow the agarose to solidify and then were placed in lysis buffer and incubated for 30 min at 4 °C in the dark. Denaturation was achieved by transferring the slides to a pre-chilled alkaline electrophoresis buffer (300 mM sodium hydroxide, 1.0 mM EDTA) and incubating for 30 min at 4 °C in the dark. The slides were then transferred into a horizontal electrophoresis chamber (BioRad Inc., Hercules, CA) and subjected to electrophoresis at 1 V/cm (30 V, 300 mA) for 15 min. Following electrophoresis, slides were washed 3 times with water, once with 70% ethanol, and air dried. The dried slides were stained with 100 μL of diluted Vista Green DNA dye (Cell Biolabs Inc., San Diego, CA), and cellular DNA was visualized using a FITC filter-fitted microscope. Comet images were scored visually on a scale of 0 to 4 as described by Collins [23], with 0 representing no damage and 4 representing severe damage.

### ELISA

To generate ASC-conditioned media, we cultured fourth passage ASCs in complete media until confluence, and the old media was replaced with fresh. After 3 days, conditioned media were collected and centrifuged at 300 g for 5 min. The resulting supernatants were frozen. The cells were also collected and the total number counted prior to protein extraction and quantification. IL-6, MCP-1, TNF-alpha, and IL-1β were measured using ELISAs purchased from R&D Systems, Inc. (Minneapolis, MN).

### Western Blot Analysis

Protein was isolated from ASCs at varying times using protein lysis buffer composed of 2 mL 5X lysate buffer (750 mM sodium chloride, 250 mM Tris, 5% Triton X100, 5 mM EDTA), 1 pill of protease inhibitor (Roche, Basel, Switzerland), 100 μL of phosphatase inhibitor cocktail (Sigma, St. Louis, MO), and 66.7 μL of phenylmethylsulfonyl fluoride in 8 mL of water. Total protein concentration was measured with the BCA protein assay as per manufacturer’s instructions (Pierce, Rockford, IL). Following protein measurement, around 40 μg of protein was quenched in buffer composed of 400 mM Tris (pH 6.8), 40% glycerol, 1% bromophenol blue, and 0.8 mL of β-mercaptoethanol in 10 mL of water. The samples were resolved on a sodium dodecyl sulfate polyacrylamide gel (Bio-Rad, Hercules, CA) at 150 V for 1 h. The proteins were then transferred onto a PVDF membrane for 90 min at 400 mA. Protein transfer to the membrane was confirmed by Ponceau staining. The membrane was incubated in 3% BSA for 1 h at the room temperature and incubated with a primary antibody to NF-κB (Abcam, Cambridge, MA; 1:1000) at 4 °C overnight. The blot was washed three times with Tris Buffered Saline s-Tween 20 for 10 min and incubated with HRP-conjugated anti-rabbit secondary antibody (1:5000) for 1 h at room temperature. The proteins were visualized using Enhanced chemiluminescence Fast Western blotting substrate (Thermo Scientific-Pierce, Rockford, IL) per the manufacturer’s instructions. The membranes were then stripped and re-blotted with an anti-GAPDH rat antibody (Cell Signaling Technology, Danvers, MA; 1:1000) for 1 h at room temperature and HRP-conjugated anti-rabbit secondary antibody (Jackson ImmunoResearch Laboratories, Inc., West Grove, PA; 1:5000) for 1 h at room temperature. The intensity of band was measured using the Image J 1.47 software package (National Institutes of Health, imagej.nih.gov/ij/download/).

### Statistical Analysis

Analysis of variance with Tukey’s test and paired or unpaired Student’s *t* tests were used as appropriate. Data were expressed as the mean ± standard error of the mean, as indicated. Significance was accepted at *p* < 0.05.

## Results

### The SVF and Adipocytes Produce Mediators of Inflammation Following Burn Injury

**Fig. 1:**
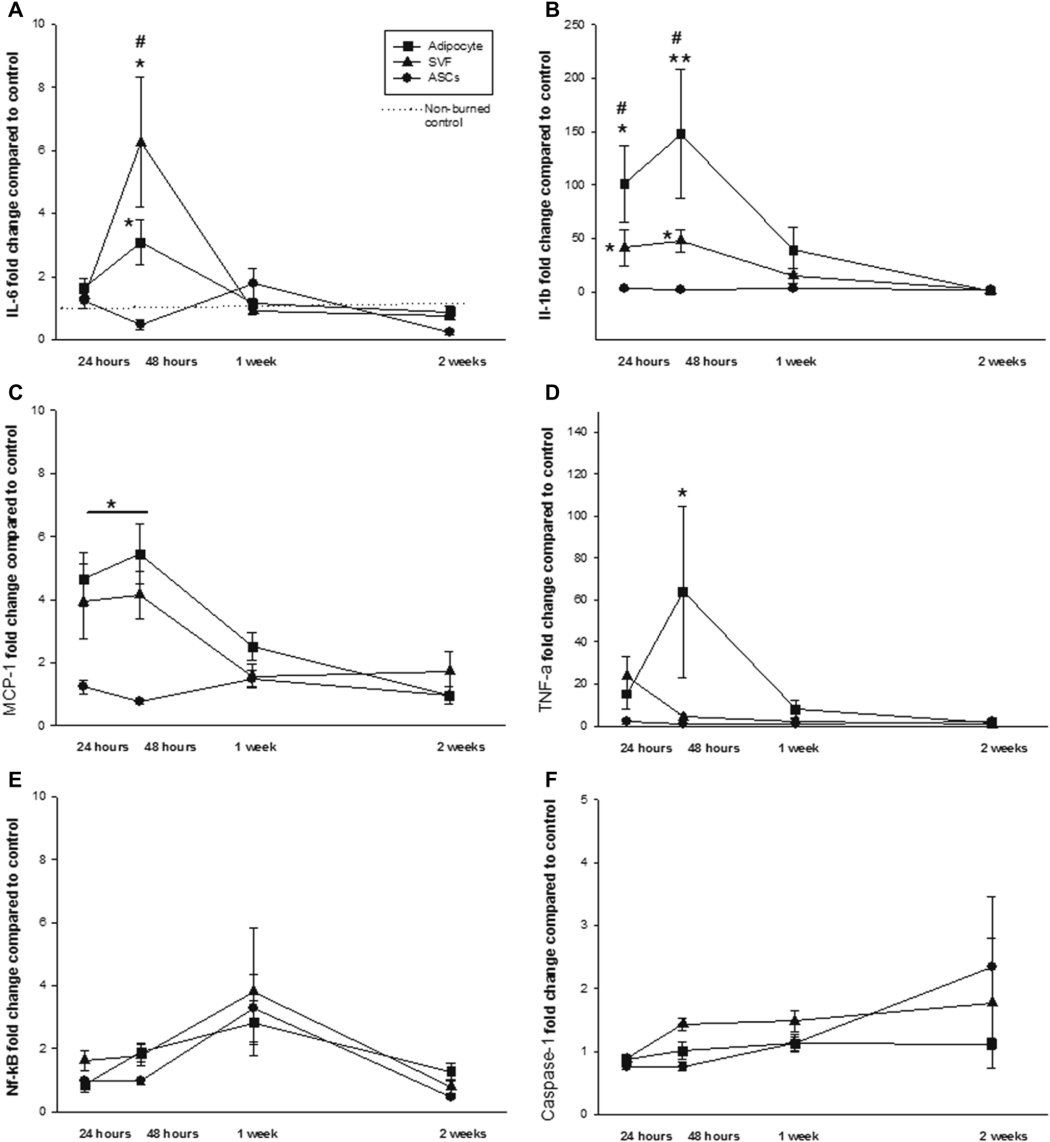
Effect of burn injury on cytokine and transcription factor mRNA production by adipocytes, the stromal vascular fraction (SVF), and enriched ASCs. Temporal alterations in expression of (A) IL-6, (B) IL-1β, (C) MCP-1, (D) TNF-α, (E) caspase-1, and (F) NF-κB are shown. Data points represent mean ± SEM of 8 control animals or 6 burned animals (24 h, 48 h 1, and 2 weeks postburn #p <0.05 vs. SVF, **p <0.005 vs. ASCs﻿,﻿ *p <0.05 vs. ASCs).

Messenger RNA expression of inflammatory markers (IL-1β, IL-6, MCP-1, caspase-1, TNF-α, and NF-kB) was measured in freshly isolated adipocytes, the SVF, and enriched ASCs (Fig. 1). A significant elevation in IL-1β mRNA occurred in adipocytes and the SVF at 24 and 48 h following burn injury, compared to non-burned controls, (*p* = 0.037, Fig. 1b). When compared to expression in non-burned controls, expression of IL-6 mRNA was significantly altered by burn injury (*p* = 0.009). In adipocytes, IL-6 mRNA increased while in ASCs it decreased, both at 48 h after injury (*p* = 0.009, Fig. 1b). A significant decrease in MCP-1 mRNA expression was found at 24 and 48 h postburn in enriched ASCs (*p* = 0.005, Fig. 1c). TNF-α mRNA increased significantly in adipocytes at 48 h following burn injury (*p* = 0.05, Fig. 1d). Burn injury did not induce changes in expression of caspase-1 or NF-κB mRNA in any of the cell types, regardless of the time point (Fig. 1e, f). In ASCs, protein levels of IL-6, MCP-1, TNF-α, IL-1β, and NF-κB were unaffected by burn injury (data not shown).

### DNA Damage Appears in the SVF Soon after Burn Injury but Resolves by 72 Hours Post Injury

**Fig. 2:**
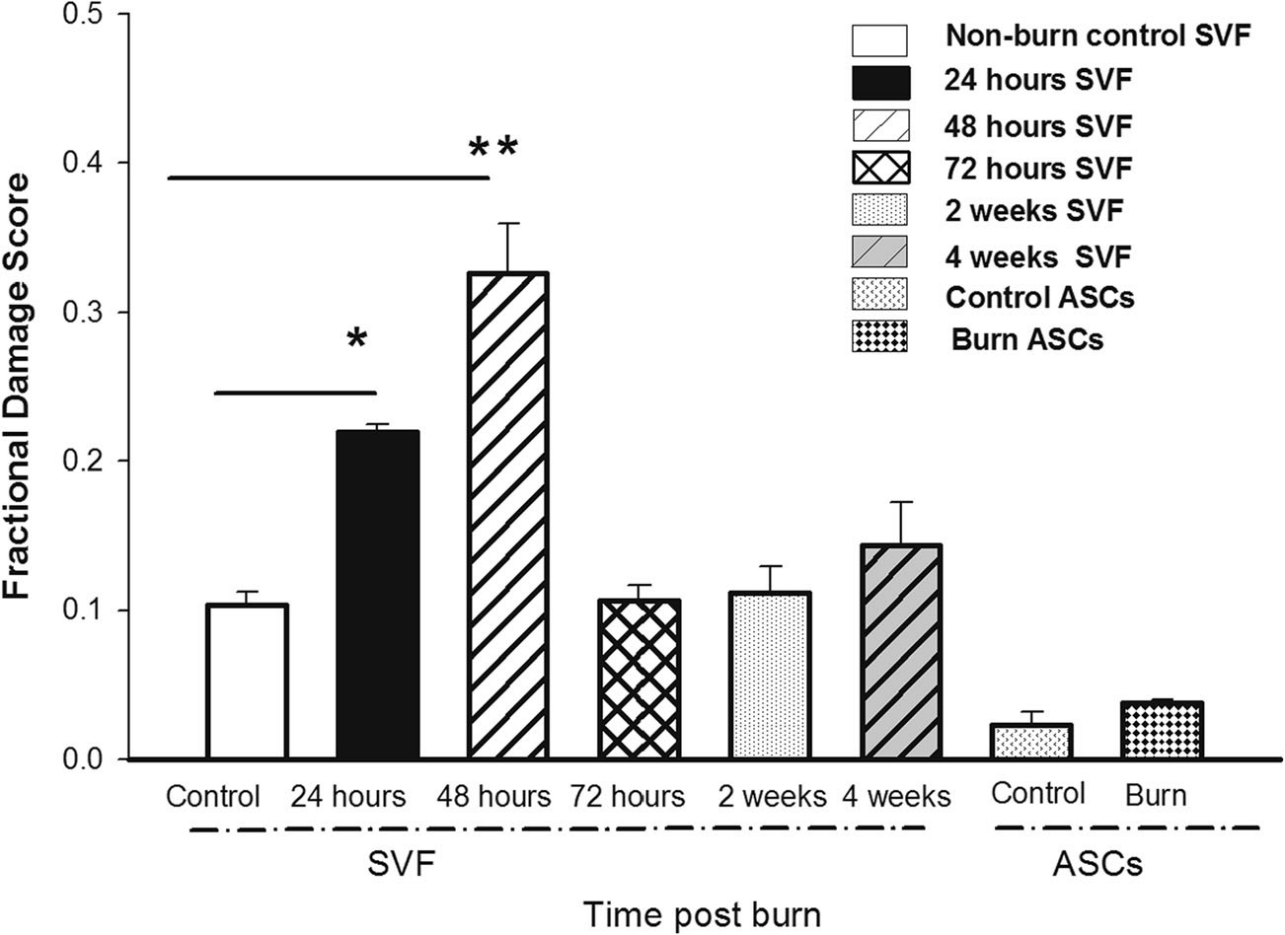
Burn injury induces minimal DNA damage in the stromal vascular fraction (SVF) and enriched ASCs. Each bar represents the mean ± SEM of 8 control animals or 6 burned animals (24, 48, or 72 h, 1, 2, and 4 weeks postburn). *p < 0.05 and **p < 0.005 vs. control

DNA damage to the cells in the SVF and the enriched ASCs was assessed by comet assay. There was a significant induction of DNA damage in SVF isolated 24 and 48 h postburn (*p* = 0.05, *p* = 0.005, respectively) (Fig. 2) compared to non-burned control. This amount of damage correlated to 4 damaged cells per 100 isolated cells. This damage resolved by 72 h postburn. In cultured ASCs, the level of damage remained the same throughout the 4-week experimental period. Burn injury and subsequent culturing of the ASCs did not induce DNA damage.

### Burn Injury Does Not Alter the Differentiation Potential of ASCs

**Fig. 3:**
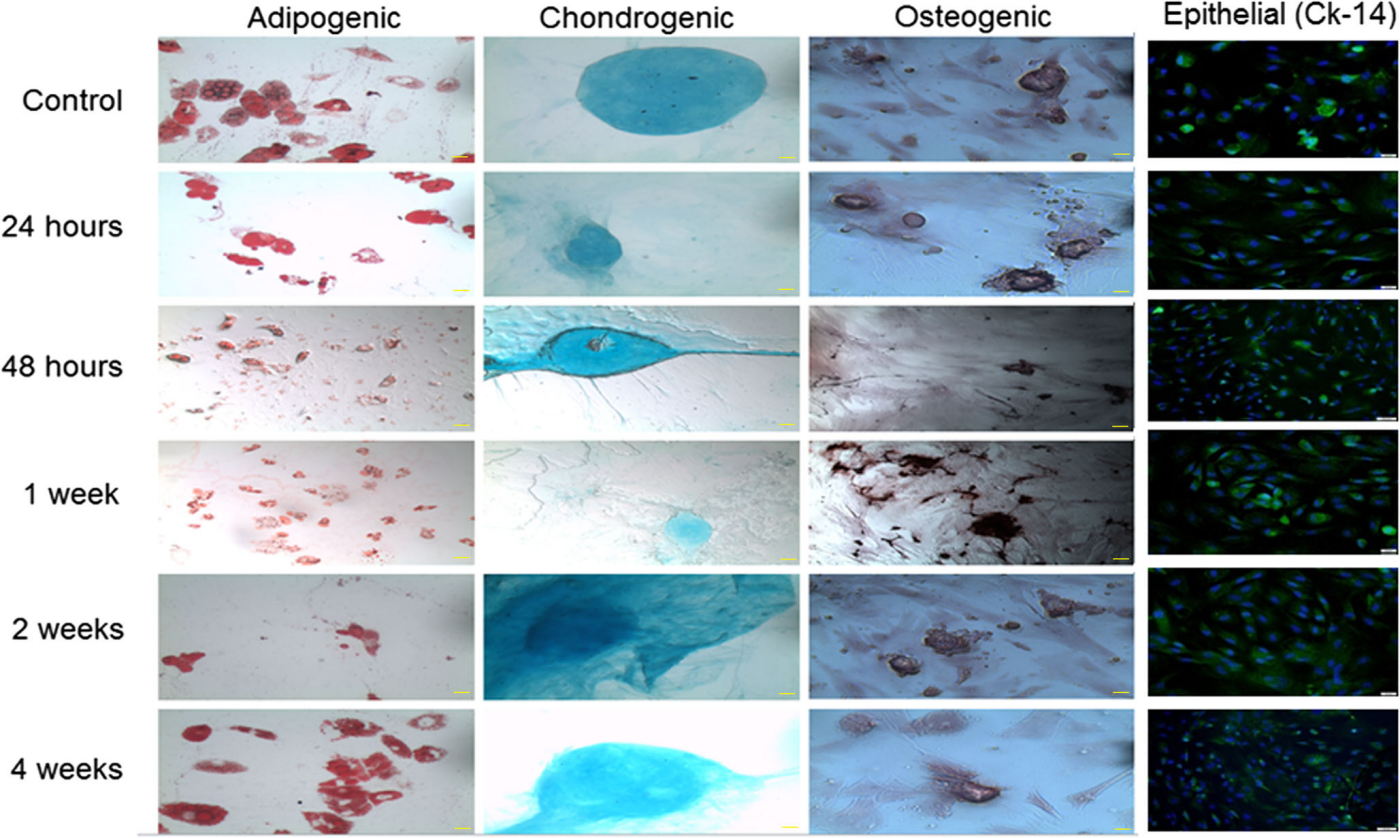
Burn injury does not alter the ability of the ASCs to differentiate into adipogenic, chondrogenic, osteogenic, or epithelial lineages. Differentiated ASCs were identified by staining with oil-O-red (adipogenic cells), alcian blue (chondrogenic cells), or alizarin red (osteogenic cells) or by immunostaining for CK-14 (epithelial cells; green) and counterstaining nuclei with DAPI (blue). Images are shown at 10× magnification, with 50 μM scale bar at the bottom right

**Fig. 4:**
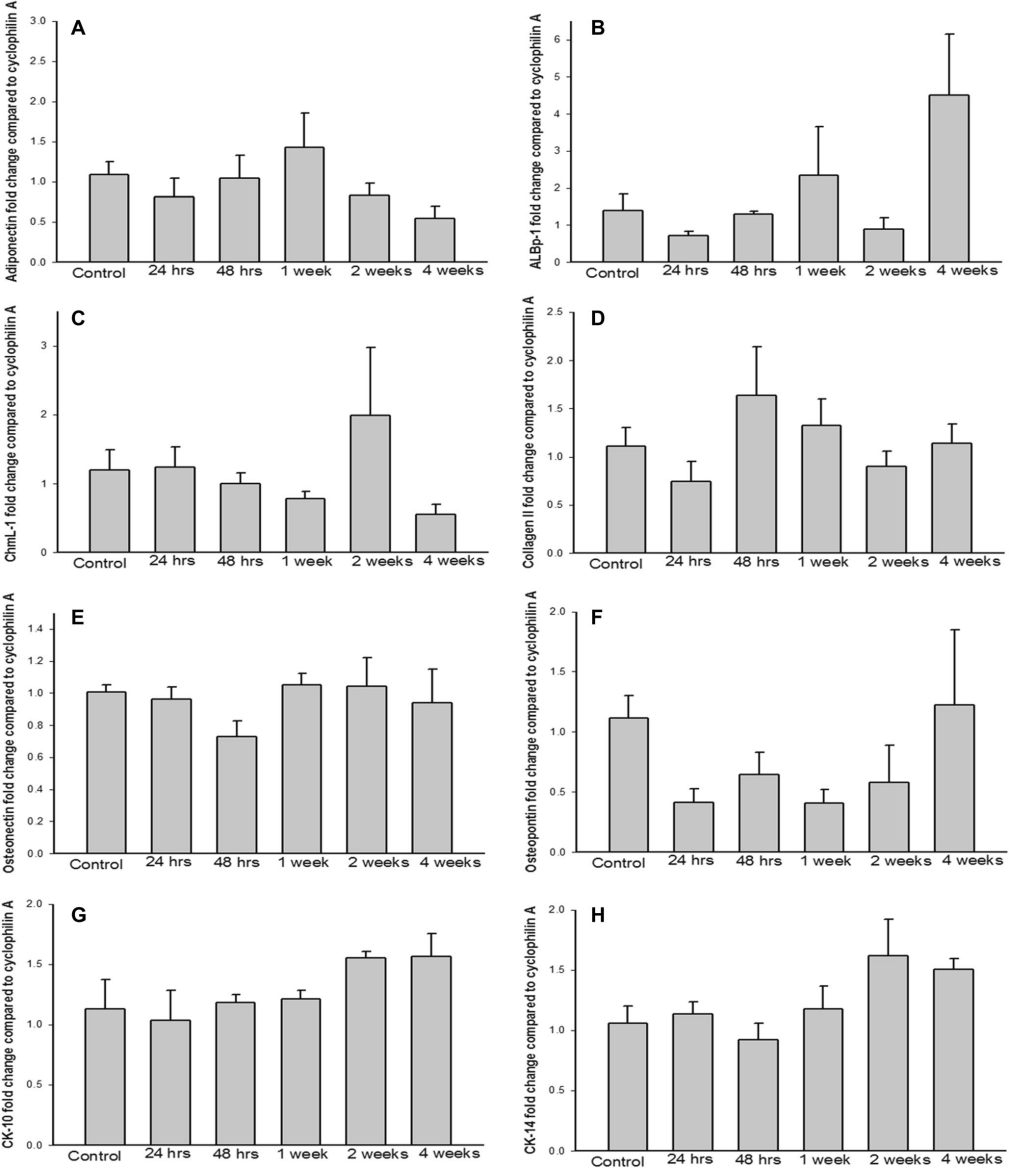
Burn injury does not affect expression of cell type-specific genes in differentiated ASCs. Messenger RNA levels of (**a**) adiponectin and (**b**) adipocyte lipid binding protein 1 in adipogenic cells, (**c**) chondromodulin I and (**d**) collagen II in chondrogenic cells, (**e**) osteonectin and (**f**) osteopontin in osteogenic cells, and (**g**) cytokeratin 10 and (**h**) cytokeratin 14 in epithelial cells. Each bar represents the mean ± SEM of 8 control animals or 6 burned animals (24 and 48 h, 1, 2, and 4 weeks postburn)

Following isolation and enrichment, ASCs were cultured in media formulated to induce differentiation into adipocytes, osteoblasts, chondrocytes, or epithelial cells. Differentiation into each of these cell types was confirmed by staining with oil O red (adipocytes), alizarin red (osteocytes), or alcian blue (chondrocytes) or by immunofluorescence staining for cytokeratin-14 (epithelial cells) (Fig. 3). ASCs from burn animals retained their differentiation capacity at all time points examined. The abundance of mRNA specific to each of the differentiated cell types was also measured. As shown in Fig. 4, we detected no significant differences between differentiated ASCs from non-burned and burned animals in levels of mRNA encoding factors involved in adipogenesis, (adipocyte lipid binding protein-1 and adiponectin [24]), chondrogenesis (chondromodulin 1 and collagen II [25, 26]), osteogenesis (osteopontin and osteonectin [27]), and epithelial differentiation (cytokeratin [CK]-10 and CK-14).

### Postburn ASC Populations, as Identified by CD Marker Expression, Are Stable

**Fig. 5:**
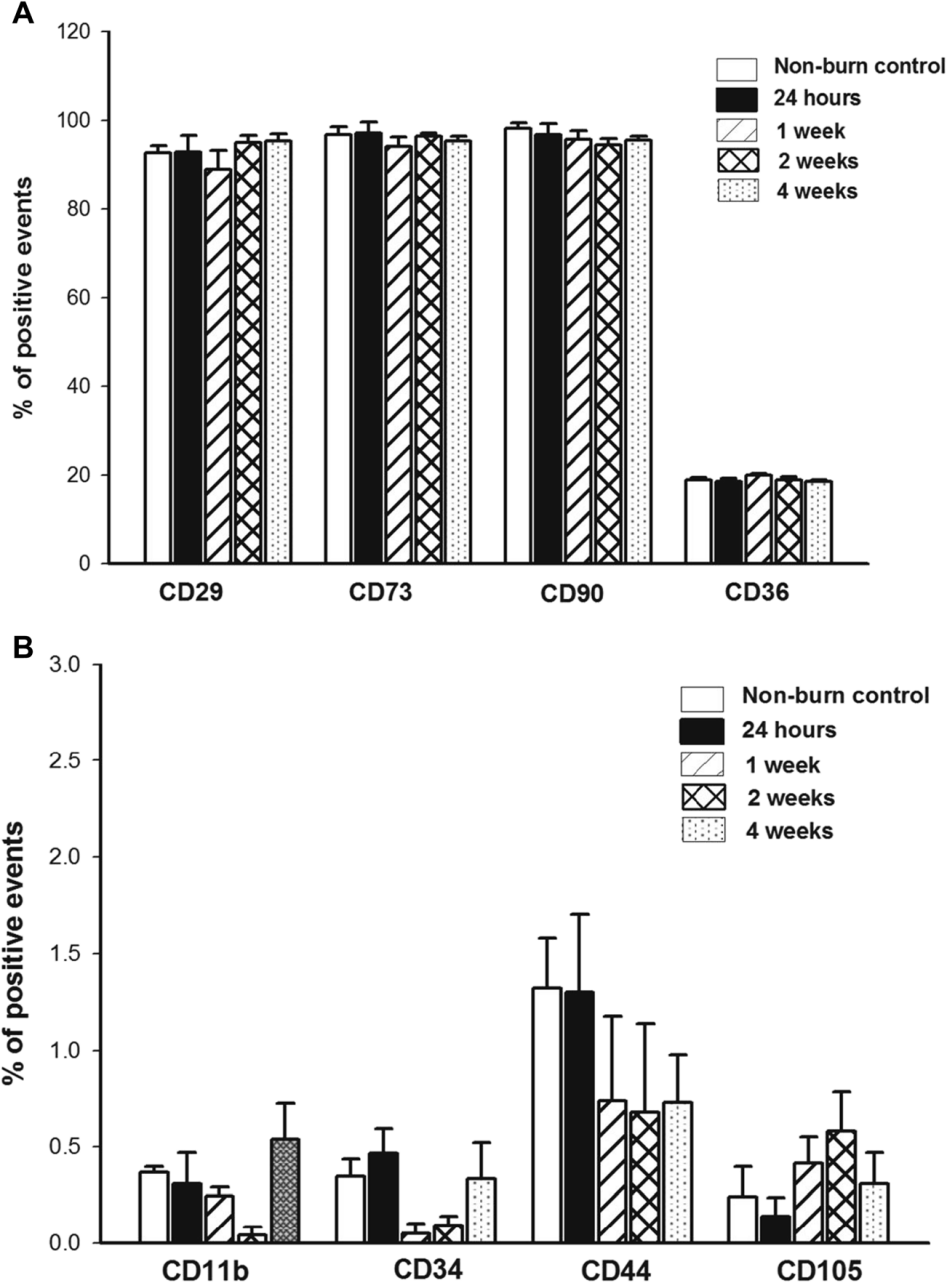
The ASC population is stable following burn injury, as confirmed by CD marker protein levels. No differences were detected in (**a**) CD29, CD73, CD90, or CD36 (**b**) or in CD11b, CD34, CD44, or CD105. Each bar represents the mean ± SEM of 8 control animals or 6 burned animals (24 h, 1, 2, and 4 weeks postburn)

Expression of cell-surface and intracellular CD markers was assessed by flow cytometry. There were no significant differences in the expression of protein levels of CD11b, CD34, CD44, CD105, CD29, CD73, CD90, or CD36 (Fig. 5a, b). Similarly, there were no differences in the abundance of mRNA encoding these markers (data not shown).

### ASC Proliferation Is Not Affected by Burn Injury

**Fig. 6:**
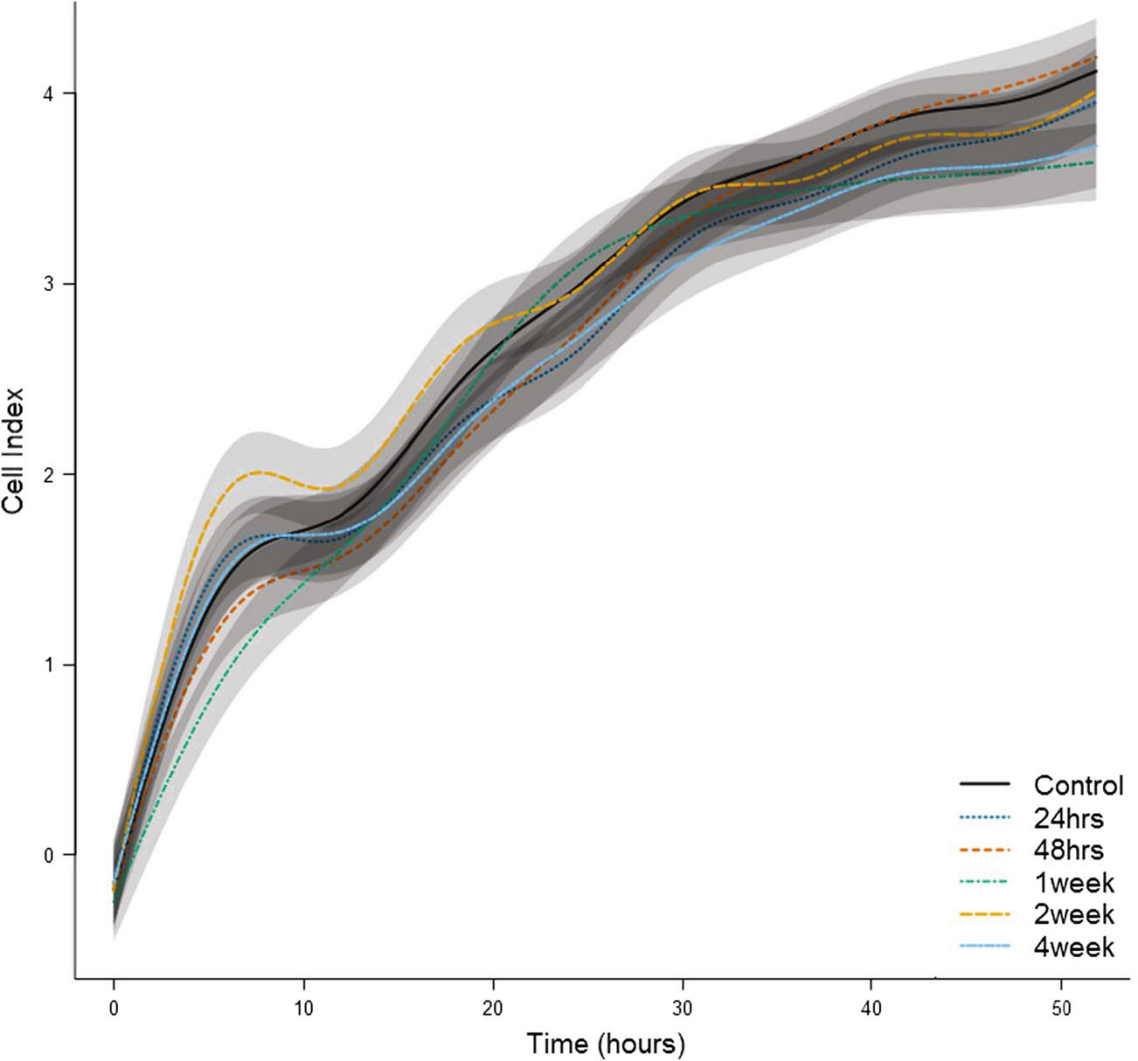
Burn injury does not affect proliferation of ASCs. Lines are averages of continuous measurements, and shaded regions around each line indicate SEM. Eight animals were included in the control group and 6 in the burned group (24, 48 h, 1, 2, and 4 weeks postburn)

Measurement of cell proliferation over a 50-h period via cell impedance showed that cell proliferation was not altered by burn injury (Fig. 6).

## Discussion

Here we have shown that post-burn inflammation in the adipose tissue is mediated by the adipocytes and the SVF. Furthermore, we have shown that the effect of burn injury on the ASCs is relatively small. ASCs can be isolated in great abundance, can differentiate into multiple cell types, and are believed to be safe for autologous and allogeneic transplantation [28]. Because of these qualities, ASC-based therapies have become one of the most promising approaches in the treatment of myriad diseases [3, 5, 28]. As it is possible to utilize a patient’s own ASCs for repair and regeneration following injury or disease, characterization of how these insults affect stem cells that may be utilized for therapeutic purposes is of paramount importance. Inflammation occurs in adipose tissue following injuries such as burns or in association with diseases such as metabolic syndrome or diabetes. By altering the inflammatory milieu where the ASCs reside, it is possible that particular subpopulations of ASCs are selected to proceed toward a different fate or induced to migrate, thereby depleting the tissue of specific subpopulations of ASCs and changing the overall ASC population that could be isolated for clinical use. Therefore, the purpose of our study was two-fold: 1) identify the cells within the adipose contributing mediators of inflammation to the response to burn injury and 2) determine the effect of burn injury on the fate and function of the ASCs. This was accomplished using the clinically relevant 60% rat scald burn model. Prior work has shown that this model induces a hypermetabolic response similar to that seen in patients with large burn injuries, including increased catabolism and inflammation [21]. Following isolation of the adipose tissue, we were able to obtain adipocytes and SVF for immediate study as well as enriched ASCs using a standard isolation protocol that yielded ASCs for study 48 h later. These studies show that inflammation comes from adipocytes and the SVF and that ASCs are not inflammatory in culture (nor are they affected by the burn injury and subsequent culturing procedures).

Stem cell fate and function are predominantly determined by dynamic interactions between the stem cells and their environment. Stem cells interact with the extracellular matrix, neighboring cells, and secreted proteins, allowing changes in the microenvironment to affect stem cells [18, 19, 29]. In adipose tissue, ASCs and adipocytes are surrounded by factors that facilitate tissue homeostasis under normal conditions. Following bury injury, many pro- and anti-inflammatory cytokines are elevated both systemically and locally within burned and non-burned tissue [20]. In freshly isolated adipocytes and SVF, we showed that IL-1β and IL-6 are elevated in response to burn injury but that levels return to those seen under non-burn conditions between 48 h and 1 week following the injury. Similarly, TNF-α was elevated in adipocytes but returned to normal levels after burn injury within the same temporal window. We are unable to measure immediate levels of inflammatory mediators in the ASCs, as the isolation protocol necessitates a 48-h incubation period. Therefore we measured the same markers in enriched, post-isolation ASCs that were cultured in vitro for 48 h. Under these conditions, ASCs from burned animals produced similar levels of inflammatory markers as those from non-burned animals. An additional finding of interest was that the mature adipocytes produced significantly greater amounts of inflammatory markers than the SVF, which contains macrophages and other inflammatory cells. Within adipose tissue, ASCs, mature adipocytes, and other cell types likely function through paracrine signaling [30–32]. These interactions should be studied further to determine the role of this inflammatory response following burn injury. Additionally, we do not know whether increased DNA damage results in greater cell death within adipose tissue, leading to further increases in the local inflammatory response or whether this damage is repaired. The comet assay was performed to evaluate whether ASCs obtained following burn injury had increased DNA damage. Significantly higher fractional damage scores were recorded in the SVF isolated at 24 and 48 h postburn than in the non-burn control SVF. Whether these amounts of DNA damage are sufficient to affect tissue function or lead to aberrant cell behavior is unknown.

We were able to isolate ASCs from animals with large burn injuries. ASC identity was confirmed by measuring stemness markers; by differentiating the cells into osteogenic, chondrogenic, adipogenic, and epithelial cells [33]; and by verifying terminal differentiation into these cell types through measurement of cell type-specific gene expression. Burn injury did not change the ability of the ASCs to differentiate into other cell types. It also did not affect ASC proliferation or expression of mRNA for cytokines, growth factors, or CD markers related to burn injury.

Here we used the consensus definition to identify ASCs [33, 34]. Most published studies have shown that CD13, CD90, CD73, CD29, CD36, and CD105 are positive markers for ASCs, while CD34 and CD11b/c are negative markers. Low expression of CD105 expression in rat ASCs could be mainly attributed due to lack of appropriate anti-rat CD105 antibody or an inadequate affinity of the CD105 antibody for the rat homologue as opposed to murine or human CD105. It may also be attributable to down regulation or loss of CD105 in culture [35].

In conclusion, ASCs obtained in a model of severe burn injury behave similarly to ASCs obtained from non-burned controls. Given the fact that inflammatory markers are elevated and DNA damage is increased 24 to 48 h postburn, we recommend that ASCs or SVF be used for tissue engineering or wound healing applications in severely burned patients after inflammation resolves. Confirmatory studies utilizing human tissue are needed to determine the timeline for resolution of the inflammation.
